# Risk factors for predicting symptomatic adjacent segment degeneration requiring surgery in patients after posterior lumbar fusion

**DOI:** 10.1186/s13018-014-0097-0

**Published:** 2014-10-12

**Authors:** Jinqian Liang, Yulei Dong, Hong Zhao

**Affiliations:** Department of Orthopaedic Surgery, Peking Union Medical College Hospital, No. 1 Shuai Fu Yuan, Wang Fu Jing Street, 100730 Beijing, China

**Keywords:** Lumbar, Adjacent segment degeneration, Fusion, Risk factor

## Abstract

**Background:**

Although measures to reduce and treat degenerative changes after fusion are discussed, these are still controversial.

**Methods:**

A retrospective study was conducted on a consecutive series of 3,799 patients who underwent posterior lumbar fusion for degenerative lumbar disease between January 1999 and January 2009. A total of 28 patients with symptomatic adjacent segment degeneration surgery were identified. Another group of 56 matched patients with degenerative lumbar disease without symptomatic adjacent segment degeneration after spinal fusion were marked as the control group. These two groups were compared for demographic distribution and clinical and radiographic data to investigate the predictive factors of symptomatic adjacent segment degeneration surgery by logistic regression.

**Results:**

The overall incidence rate of symptomatic adjacent segment degeneration surgery was 0.74%. Strong risk factors for the development of a symptomatic adjacent segment degeneration requiring surgery were preoperative distance from L1 to S1 sagittal plumb line (p = 0.031), preoperative lumbar lordosis (p = 0.005), and preoperative adjacent disc height (p = 0.003). Mean postoperative lumbar lordosis was smaller (p = 0.000) in symptomatic adjacent segment degeneration surgery (SASDS) group compared with in the control group (33.3° vs. 39.8°). Postoperative adjacent disc height was also significantly lower in the former group compared with the latter group (p = 0.002). Logistic regression analysis showed that body mass index (BMI) (OR: 1.75; p = 0.006), preoperative adjacent disc degeneration (ADD) on MRI (OR: 13.52; p = 0.027), and disc bulge in preoperative CT examination (OR: 390.4; p = 0.000) maintained their significance in predicting likelihood of symptomatic adjacent segment degeneration surgery.

**Conclusions:**

The occurrence of a symptomatic adjacent segment degeneration surgery is most likely multifactorial and is related to BMI, preoperative ADD on MRI, and disc bulge in preoperative CT examination.

## Introduction

Posterior lumbar fusion with pedicle screw fixation has been used widely in such settings as trauma, disc herniation, spondylosis, and tumors because of the advantages pedicle screw fixation offers: initial stability, higher fusion rate, and recovery of normal lordosis while sparing the necessity for external support. In addition, good results with this technique have been reported in literature. However, in spite of the procedure’s success at achieving successful fusion in spinal surgery, a long-term follow-up after a solid fusion has revealed degenerative changes including segmental instability, spinal stenosis, intervertebral disc lesion, retro-spondylolisthesis, and fracture at the adjacent segments [1-3]. Although the development of adjacent segment degeneration can be considered as a part of the normal aging and degenerative process, this phenomenon appears to be at least partly influenced by the altered stresses that arise as a consequence of lumbar fusion [4-7]. Measures to reduce and treat degenerative changes after fusion are discussed, along with the increased interest in causative factors related to the prevention and treatment reported by many studies. Nevertheless, these are still controversial. Therefore, the identification and quantification of risk factors for symptomatic adjacent segment degeneration requiring surgery in patients after posterior lumbar fusion are of paramount importance to the patient and the clinician. In addition to its obvious importance for patient safety, risk factor information becomes critical as health care policy makers implement and enforce ‘quality’ metrics.

This retrospective cohort study was undertaken to investigate 1) the overall incidence of adjacent segment degeneration in a large population of patients with a background degenerative lumbar condition treated with spinal fusion and instrumentation and 2) the predictive factors for the development of symptomatic adjacent segment degeneration requiring surgery in patients after posterior lumbar fusion.

## Materials and methods

We examined data from a consecutive series of 3,799 patients who underwent posterior lumbar fusion for degenerative lumbar disease (back pain symptoms attributable to intervertebral disc degeneration that includes pathologic changes in the disc, annulus, and the end plates, with or without osteophyte formation at the vertebral apophyses) between January 1999 and January 2009, at one academic hospital—a university-based medical center. Those patients undergoing surgery for nondegenerative disease (trauma, infection, tumor, deformity, inflammation) were excluded. Criteria of degenerative change at adjacent segments: 1) Anterior or posterior displacement of >3 mm was found on the X-ray of the sagittal plane of the closest upper segment and the closest lower segment at the last follow-up. 2) The height of the intervertebral disc relative to that of the upper interbody had declined by 20%. 3) A segmental motion instability of more than 15° was observed on the X-ray of the sagittal plane with flexion and extension. Those patients who had symptomatic adjacent segment degeneration were defined as degeneration at a segment adjacent to a fusion-causing symptoms. The cohort identified was divided into patients undergoing symptomatic adjacent segment degeneration surgery (SASDS) and those who did not (control). The SASDS group was composed of patients who underwent secondary adjacent segment surgery because of symptoms concordant with adjacent segment pathology. Only those patients who had a minimum of five-year follow-up were reviewed. All patients with SASDS were identified, and these patients in the control group were chosen and best matched for sex, age, approximate date of surgery and diagnosis for the patients with SASDS, and then matched at a 2:1 ratio, respectively.

Demographic data included age at primary surgery, gender, smoking history, body mass index (BMI), and diagnosis. Follow-up was defined as the time from primary surgery to reoperation in the SASDS group and the period after surgery in the control group. Surgical data collected included levels of surgery, number of fusion levels, and type of bone graft (autograft vs. allograft). Radiographs at the primary surgery and at the final follow-up or before an additional surgery were assessed in each group to determine the lumbar lordotic angle, the distance between the L1 and the S1 sagittal plumb lines, the sagittal slope angle of the superior end plate of S1, adjacent disc angle, and adjacent disc height. In addition, magnetic resonance imaging (MRI) was used to investigate whether there had been a degree of preoperative adjacent disc degeneration (ADD). Patients shown to have grade ≥III in the five-grade classification of Pfirrmann et al. [8] based on MRI were considered to have a degenerative change.

**Figure 1:**
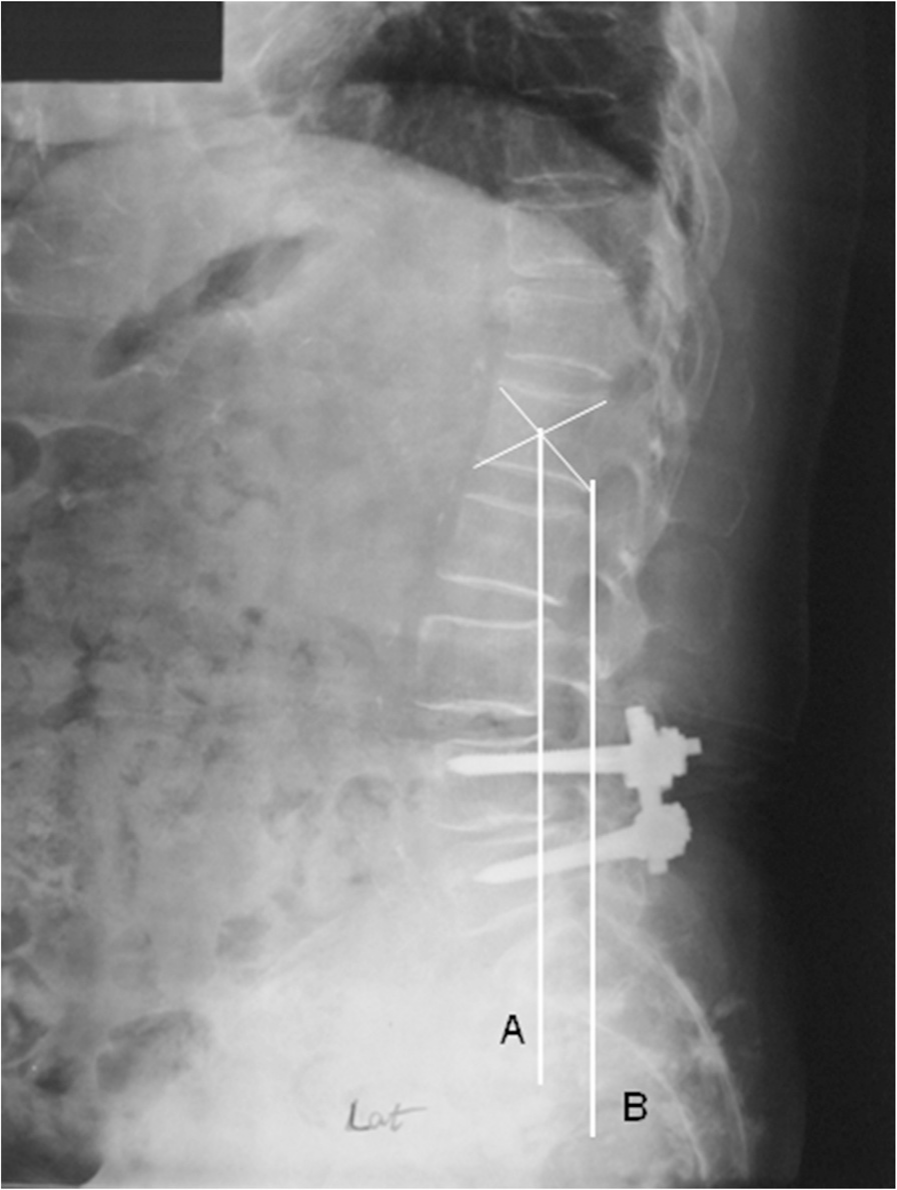
**Radiographs were assessed to determine distance from L1 to S1 sagittal plumb line (A–B).**

**Figure 2:**
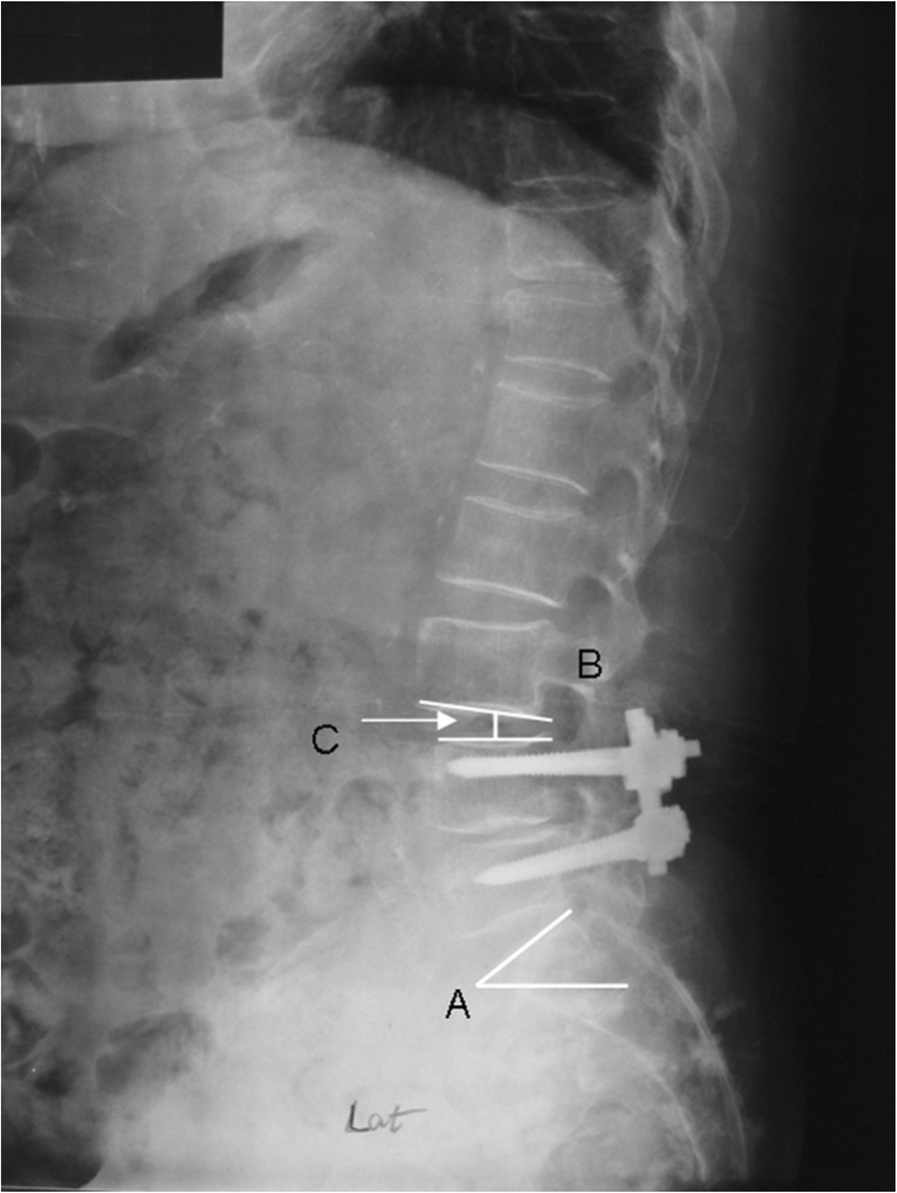
**Radiographs assessed for slope angle, disc angle, and disc height.** Radiographs assessed for S1 sagittal slope angle **(A)**, adjacent disc angle **(B)**, and adjacent disc height **(C)**.

Routine lateral radiographs were obtained using standard techniques. The patient stands upright, his or her head facing forward. The X-ray tube is positioned 72 in from the patient. The lumbar lordotic angle was measured by Cobb’s angle made by the upper endplate of the first lumbar vertebra and the upper endplate of the sacrum. The L1 sagittal plumb line was drawn with a lateral gravity plumb line from the center of L1 (Figure 1). The center of L1 was noted by the intersection of crossing diagonals of vertebral body of L1 on the lateral radiograph. The S1 sagittal plumb line was drawn with a lateral gravity plumb line from the posterior end of S1 vertebrae (Figure 1). The distance between the plumb lines was measured as the shortest perpendicular distance between the two lines (Figure 1). The sagittal slope angle of the superior end plate of S1 was measured as the angle between a horizontal line and the superior end plate of S1 (Figure 2). The adjacent disc angle was measured as the angle between the caudal and cranial end plates of the disc just adjacent to the upper or lower instrumented/fused levels (UIV or LIV) (Figure 2). The adjacent disc height was measured on lateral radiograph from the middle of the superior border of the disc to the middle of the inferior border of the disc just adjacent to the upper or lower instrumented/fused levels (UIV or LIV) (Figure 2).

Factors associated with SASDS were identified using univariate analysis. The data analysis was performed using SPSS version 19.0 (Chicago, IL, USA). Continuous data were compared between the two groups using the student *t* test, whereas discontinuous data were analyzed using the chi-squared test. Fisher’s exact test was used for small data subsets (n < 5). All significance tests were two-tailed, with p <0.05 representing statistical significance. In addition, a multivariate logistic regression analysis was performed to identify which factors helped predict the probability of a SASDS.

## Results

### Demographic data

A summary of the clinical data before spinal fusion for the SASDS and control groups is presented in Table 1. Of the 28 patients identified as having SASDS, 3 had disc herniation, 3 had lumbar spondylolisthesis, and 22 had lumbar stenosis. The other group of 56 patients without SASDS matched for age, diagnosis, and year of surgery also were evaluated: 12 had disc herniation, 4 had lumbar spondylolisthesis, and 40 had lumbar stenosis. Mean age at index surgery was 61.4 years (range, 34–79 years) for the group of patients who subsequently developed symptomatic adjacent segment degeneration requiring surgery and 62.1 years (range, 38–78 years) for the other matched cohort. Symptomatic adjacent segment degeneration requiring surgery was observed in 0.74% (28 of 3,799) of patients, and the average symptom-free period was 5.66 years (range, 2.8–10.2). The distribution was as follows: superior adjacent level, 15 patients (54.6%) with a symptom-free period of 5.71 years; inferior adjacent level, 10 patients (35.7%) with a symptom-free period of 5.45 years; both levels, 3 patient (10.7%) with a symptom-free period of 6.15 years.

**Table 1:** **Demographic characteristics and surgery-related factors of the patients**

**Characteristics**	**Patients with SASDS**		***P*** **values**
	**Yes (** ***n*** **= 28)**	**No (** ***n*** **= 56)**	
Age (years)	61.4 ± 12.0	62.1 ± 10.3	0.767
Sex, *n* (%)			0.516
*Male*	11 (39.3)	18 (32.1)	
*Female*	17 (60.7)	38 (67.9)	
BMI (kg/m^2^)	27.9 ± 2.3	25.2 ± 3.3	0.000
Smoker within past year, *n* (%)			0.259
*Yes*	8 (28.6)	10 (17.9)	
*No*	20 (71.4)	46 (82.1)	
Diagnosis, *n* (%)			0.446
Disc herniation	3 (10.7)	12 (21.4)	
Lumbar spondylolisthesis	3 (10.7)	4 (7.1)	
Lumbar stenosis	22 (78.6)	40 (71.4)	
No. of levels fused	1.50 ± 0.69	1.57 ± 0.63	0.637
Fusion method, *n* (%)			0.577
*PLF*	25 (89.3)	52 (92.9)	
*PLIF*	3 (10.7)	4 (7.1)	
Allograft, *n* (%)			0.271
*Yes*	19 (67.9)	31 (55.4)	
*No*	9 (32.1)	25 (44.6)	

Note: SASDS symptomatic adjacent segment degeneration surgery, ASD adjacent segment degeneration, BMI body mass index, PLIF posterior lumbar interbody fusion, PLF posterior lumbar fusion.

The average body mass index (BMI) for the SASDS and control groups was 27.9 ± 2.3 and 25.2 ± 3.3, respectively (p = 0.000). No significant differences were observed between the two groups in age, sex ratio, number of fused segments, or the fusion method.

### Radiographic outcome

The preoperative MRI and CT of L1–S1 levels were measured preoperatively; Of the 28 patients with SASDS, 16 patients (57.6%) were noted to have a degenerative change. However, in the control group, only 16 patients (28.6%) were noted to have a degenerative change preoperatively (p = 0.011). The disc bulge present in preoperative CT examination was also statistically associated with increased risk of SASDS: It was found in 27 of 28 in the group with SASDS, as compared with 8 of 56 in the matched cohort (χ^2^ p = 0.000).

The following radiographic findings did not differ significantly between the two groups: preoperative S1 sagittal slope angle, preoperative adjacent disc angle, postoperative distance from L1 to S1 sagittal plumb line, postoperative S1 sagittal slope angle, and postoperative adjacent disc angle (Table 2). Strong risk factors for the development of a symptomatic adjacent segment degeneration requiring surgery were preoperative distance from L1 to S1 sagittal plumb line (p = 0.031), preoperative lumbar lordosis (p = 0.005), and preoperative adjacent disc height (p = 0.003) (Table 2). Mean postoperative lumbar lordosis was smaller (p = 0.000) in SASDS group compared with in the control group (33.3° vs. 39.8°). Postoperative adjacent disc height was also significantly lower in the former group compared with the latter group (p = 0.002).

**Table 2:** **Radiologic data for patients**

**Characteristics**	**Patients with SASDS**		
	**Yes (** ***n*** **= 28)**	**No (** ***n*** **= 56)**	***P*** **values**
Preoperative ADD on MRI, *n* (%)			0.011
*Yes*	16 (57.1)	16 (28.6)	
*No*	12 (42.9)	40 (71.4)	
Disc bulge in preoperative CT examination, *n* (%)			0.000
*Yes*	27 (96.4)	8 (14.3)	
*No*	1 (3.6)	48 (85.7)	
Preoperative distance from L1 to S1 sagittal plumb line (mm)	22.8 ± 16.5	14.2 ± 17.0	0.031
Preoperative lumbar lordosis (°)	34.4 ± 14.4	43.0 ± 12.2	0.005
Preoperative S1 sagittal slope angle (°)	33.6 ± 10.4	34.8 ± 7.5	0.540
Preoperative adjacent disc height (mm)	8.8 ± 2.5	10.4 ± 2.2	0.003
Preoperative adjacent disc angle (°)	9.5 ± 4.7	10.1 ± 4.4	0.572
Postoperative distance from L1 to S1 sagittal plumb line (mm)	17.6 ± 18.1	14.5 ± 14.0	0.384
Postoperative lumbar lordosis (°)	33.3 ± 11.4	39.8 ± 10.4	0.010
Postoperative S1 sagittal slope angle (°)	31.8 ± 9.1	34.3 ± 7.0	0.168
Postoperative adjacent disc height (mm)	8.5 ± 2.4	10.1 ± 2.2	0.002
Postoperative adjacent disc angle (°)	9.4 ± 5.7	8.9 ± 4.4	0.661

Note: SASDS symptomatic adjacent segment degeneration surgery, ASD adjacent segment degeneration.

### Predictive factors of SASDS

In the SASDS group, by multivariate logistic regression analysis, A multivariate analysis demonstrated that BMI (OR: 1.75; p = 0.006), preoperative ADD on MRI (OR: 13.52; p = 0.027), and disc bulge in preoperative CT examination (OR: 390.4; p = 0.000) maintained their significance in predicting likelihood of SASDS (Table 3). Nagelkerke R^2^ indicated that this model explained 81.9% of the variance of likelihood of SASDS.

**Table 3:** **Multivariate regression model of predicting symptomatic adjacent segment degeneration requiring surgery in patients after posterior lumbar fusion**

**Predictors**	**Odds ratio**	**95% confidence interval**		***P*** **values**
		**Lower limit**	**Upper limit**	
BMI	1.75	1.18	2.61	0.006
Preoperative ADD on MRI	13.52	1.35	135.5	0.027
Disc bulge in preoperative CT examination	390.4	21.1	7212.6	0.000

BMI body mass index, ADD adjacent disc degeneration.

Nagelkerke R^2^ = 0.819.

## Discussion

A thorough understanding of the development of clinical adjacent-segment pathology in degenerative population treated with spinal fusion and instrumentation is a critical component in avoiding revision surgery for symptomatic disease. Although previous reports have described degenerative changes adjacent to the fusion site, the predictive factors of SASDS in patients after posterior lumbar fusion have not been identified [4-6]. This study compared the patients undergoing SASDS and those who did not. The results indicate that BMI, preoperative ADD on MRI, and disc bulge in preoperative CT examination were best predictors of SASDS for patients after posterior lumbar fusion (highest ORs) rather than age, gender, diagnosis, preoperative adjacent disc angle, postoperative distance from L1 to S1 sagittal plumb line, postoperative adjacent disc angle, and other clinical or radiographic characteristics.

Many studies have focused on the altered biomechanics at the adjacent levels after fusion that result in increased mobility [9-11], increased loading [12], or increased intradiscal pressure [13], and, ultimately, accelerated disc degeneration [14-16]. This biomechanical change at the adjacent segments is affected by the range of fused segments and the sagittal angle. In a study reported by Nagata et al. [17] that involved humans and animals, the authors reported that the mobile segments adjacent to the fusion segment showed an increased range of motion, and the increase in motion at adjacent segments was in proportion to the number of the fused vertebrae. However, Soh J et al. [18] found that there was little relationship between ASD and number of fusion segments. Also, Hilibrand et al. [19] found that the risk of clinical adjacent-segment pathology after multilevel fusion was significantly less than after single-level fusion. These findings are contrary to the results expected if the biomechanical consequences of fusion were the only cause of adjacent-segment pathology. Therefore, it remains controversial whether this accelerated adjacent-level degeneration is caused by the natural progression of aging or instead by increased motion stress related to biomechanical factors secondary to surgical fusion itself. In our study, no significant differences were observed between the two groups in number of fused segments. Therefore, we think that both factors of altered biomechanical stresses and the natural history of lumbar disc disease are causative to the development of adjacent-segment pathology.

Ha KY et al. [20] reviewed some cases with preoperative intervertebral disc degeneration at the adjacent segments and found that based on MRI predictions, all the patients with over Pfirrmann grade IV developed changes in the radiographic adjacent segment. Ishihara H et al. [21] reported that the incidence of symptomatic adjacent segment disease after anterior cervical interbody fusion was higher when preoperative MRI revealed asymptomatic disc degeneration at that level. However, Soh J et al. [18] did not find any direct correlation between preoperative adjacent disc degeneration on MRI and postoperative degenerative change at the adjacent segments. In line with the studies of Ha KY et al. and Ishihara H et al., our study demonstrated that a preoperative ADD on MRI was one of the most accurate indicators for an increased risk for SASDS, suggesting that it is very important to obtain accurate information about adjacent segment before surgery and spinal fusion should be carefully considered in patients with degenerative diseases whose preoperative Pfirrmann grade in the radiographic adjacent segment is more than 3.

It has been previously suggested that alteration of sagittal plane anatomy was associated with an increased prevalence of SASDS [22,23]. Kumar at al. [24] showed that a lower incidence of adjacent-level change was demonstrated in patients with a normal C7 plumb line alignment, following lumbar spinal fusion. In the present study, a lower incidence of ASD was seen in patients with normal postoperative lumbar sagittal alignment. In addition, the preoperative distance from L1 to S1 sagittal plumb line was found to be a potential risk factor for predicting SASDS in the patients with lumbar spine fusion. A possible explanation may be that when the L1 sagittal plumb line is antepulsed, it means that pelvic compensation either did not occur (e.g. hip arthritis) or was insufficient to correct the grossly abnormal amount of antepulsion. The instantaneous axis of rotation (IAR) of a structurally normal spine passes through the anterior third of the lumbar disc spaces and the moment arm of the center of mass is balanced by the moment arm of the spinal muscles. With antepulsion, the moment arm of the center of mass increases and causes increased loading of the unfused motion segments. Another reason for disc degeneration with antepulsion is probably the extensor muscle activity during attempts to maintain balance [25]. Compressive loading of the discs is highest with trunk extension exercises [26].

The findings of this study should be viewed after considering the following limitations. Firstly, these data represent the experience at a single institution that is an academic tertiary care center with trainees in the anesthesia, orthopedic, neurosurgical, and nursing departments. Secondly, our data collection system only captured symptomatic adjacent segment degeneration information on patients who returned to our hospital for treatment. Patients with asymptomatic ASD or symptomatic ASD who were managed successfully as an outpatient would not be included in this study. However, we consider these ASDs to be clinically significant as these patients required further follow up and/or additional surgeries to treat their ASD. Moreover, the results are limited by a relatively small sample size and the broad time period covered. In addition, changes in anesthetic and surgical practices over time may have affected patient outcomes. Finally, we also acknowledge the limitations introduced by our patients’ clinical heterogeneity.

## Conclusion

The risks for SASDS for degenerative lumbar diseases are multifactorial. Multivariate logistic regression analysis suggests that BMI, preoperative ADD on MRI, and disc bulge in preoperative CT examination may be reasonable predictions for an individual likelihood of SASDS. It is our hope that surgeons might be able to improve surgical planning, advise the patient accordingly during the consent taking process, and apply strategies that would help to reduce the risk of SASDS from occurring through these predictive measures.
